# Nurse Perceptions of Artists as Collaborators in Interprofessional Care Teams

**DOI:** 10.3390/healthcare5030050

**Published:** 2017-08-30

**Authors:** Jill Sonke, Virginia Pesata, Jenny Baxley Lee, John Graham-Pole

**Affiliations:** Center for Arts in Medicine, University of Florida, P.O. Box 115900, Gainesville, FL 32611, USA; vpesata@gmail.com (V.P.); jlee@arts.ufl.edu (J.B.L.); john.gp@live.com (L.G.-P.)

**Keywords:** arts in health, arts, interprofessionalism, interprofessional collaboration, patient-centered care

## Abstract

Increased attention is being given to interprofessional collaboration in healthcare, which has been shown to improve patient satisfaction, patient safety, healthcare processes, and health outcomes. As the arts and artists are being more widely incorporated into healthcare settings throughout the world, professional artists are contributing to interprofessional care teams. A secondary directed content analysis of interviews with 31 nurses on a medical-surgical care unit investigated the roles and impacts of professional artists on the interprofessional care team. The investigation utilized established domains of interprofessional care, including values and ethics, roles and responsibilities, interprofessional communication, and teams and teamwork, and created the domain of quality of care. Findings suggest that artists are valued by nurses as members of the interprofessional care team, that they enhance the provision of patient-centered care, and that they improve quality of care by providing holistic dimensions of caring, including cognitive and social engagement, and meaningful interaction. The presence of artists on interprofessional teams provides a cost-effective and welcome resource for clinical staff and builds a culture in which creativity and interdisciplinary collaboration are more highly valued and activated.

## 1. Introduction

### 1.1. Interprofessionalism in Healthcare

Interprofessional collaboration is becoming more valued in both healthcare practice and education. The concept refers to professionals from two or more professions collaborating in a team-based approach to provide more integrated care to patients, families and communities. It implies active partnership between individuals from diverse backgrounds and professional cultures to solve problems or offer services [1,2]. Promotion of interprofessional collaboration is regarded as a priority in many health systems worldwide [3,4].

From a broader perspective, the Interprofessional Education Collaborative Expert Panel defines interprofessional professionalism as “consistent demonstration of core values evidenced by professionals working together, aspiring to and wisely applying principles of altruism, excellence, caring, ethics, respect, communication, (and) accountability to achieve optimal health and wellness in individuals and communities” [5] (p. 17). Overlapping competencies include clear communication, patient centeredness, shared values (caring and ethics) and team-based problem solving skills [6].

Within healthcare, interprofessional collaboration is also termed as interprofessional care. Studies have found that interprofessional care can improve patient satisfaction, safety, healthcare processes and outcomes and reduce death rates [7,8,9,10,11,12]. Recent studies demonstrate widespread appreciation for the concept of interprofessional care, but there is an ineffective operationalization of the concept and a need to reduce hierarchical relationships between healthcare providers [13]. Lack of interprofessional collaboration has been found to contribute to patient care errors, highlighting its importance in providing safe and effective patient care [14].

### 1.2. Arts in Health

Throughout today’s world, hospitals are recognizing the many ways in which the arts can be instrumental in the provision of patient-centered care. In the United States, there are arts programs at approximately half of accredited healthcare institutions [15]. Similar program prevalence, as well as professional networks that support this integration, exist in many regions of the world, notably the United Kingdom, Australia, Scandinavia, and Ireland.

The field of arts in health emerged organically in the 1980s, primarily in the UK and the US, as hospitals began to integrate original works of art to enhance and humanize the care environment. These programs now encompass a broad range of art forms, including visual, literary and performing arts. They enhance the aesthetics of the environment, and give patients, family members and staff valuable opportunities for enjoyment, relaxation, expression, and connection. They also demonstrate significant improvements in quality of care and specific patient outcomes [15,16,17,18,19,20,21,22].

Over the past four decades, an academic discipline has emerged to support education and professionalism in this field [23,24]. The roles and professional scope of practice of artists working in healthcare settings have been defined, clarifying that artists are neither clinicians or therapists, but that they may, by facilitating arts activities, assist clinicians in meeting clinical goals. Artists have specific knowledge and skills that allow them to use the arts to enhance the environment of care, to provide patients, family members and other healthcare workers with opportunities for enjoyment, distraction, interaction and creative expression, and to protect patient safety within their work [24,25]. To date, six peer-review journals are dedicated to research, practice and policy in arts in health, distinct from the creative arts therapies [25]. National professional associations are focusing on advocacy, policy-making, and developing strategic research agendas [20,22,26].

### 1.3. Impacts and Outcomes of Arts in Health

Numerous studies have explored the impacts of the arts within healthcare environments on patient outcomes, staff satisfaction, and the environment of care. Tuisku et al. [27] reported that cultural activities enhanced wellbeing at work among staff, and Staricoff et al. [28,29] reported that when given a choice, nurses are inclined to choose or retain employment in hospitals offering the arts. Patients report enhanced sense of control, self-awareness and social relationships, as well as reduced anxiety, in relation to participation in arts programs [30,31]. Music has been clearly shown to improve pain control, tolerance, and perception in several patient populations [32,33,34,35,36,37,38]. Studies of stress and anxiety show arts programs help reduce pain medication utilization, and the need for anesthesia and sedation [39,40,41,42,43]. Patients also experience diminished psychological and physical symptoms through relaxation and distraction [30,31,44]. The visual arts have been shown to positively impact healthcare environments by offering opportunities for non-verbal communication, and are used to affect a myriad of specific patient outcomes in oncology, cardiac, diagnostic, and long-term care settings [15,45,46,47]. Globally, health care systems are experiencing an extreme shortage of healthcare workers, including a shortage of 7.2 million nurses and shortages in 57 countries of nurses, physicians, and midwives [48]. Maintaining an adequate nursing and physician workforce has measurable economic benefits and impacts patient outcomes [48,49,50]. Health care organizations are developing strategies to retain nurses, the largest health care group in the world [48,51]. The arts have been shown to reduce nurse stress, and healthcare staff give the presence of an arts program significant consideration when seeking employment and/or remaining in current positions [28,29,52].

### 1.4. The Arts and Interprofessionalism in Healthcare

Within healthcare institutions that house arts in health programs, professional artists are increasingly utilized on interprofessional healthcare teams, often alongside creative arts therapists, who have long served in such clinical capacities. Artists most commonly work in inpatient and ambulatory oncology, pediatrics, dialysis and long-term care services. Nursing and medicine disciplines are embracing the unique knowledge and skills of professional artists to enhance unit culture, environment, and patient care. In a recent study on a medical surgical unit in an academic medical center, the specific expertise and roles of artists on the unit was viewed as an important resource to support nursing care [44].

Artists are increasingly helping interprofessional teams enhance the environment of care and add greater disciplinary breadth. This need will likely increase as regulatory bodies require documentation in the provision of patient centered care [15]. The arts bring a humanistic and holistic view to the health care team, which helps meet the mandate to provide patient-centered care. As more healthcare institutions acknowledge holistic person-centered values (i.e., recognition of the importance of caring for the whole person—mind, body, and spirit), the arts are being called upon to help provide whole-person and patient-centered care.

The arts are utilized not only within arts in health programs but also by creative arts therapists, physical and occupational therapists, recreational therapists, child life specialists, pastoral care professionals and other healthcare professionals, including nurses and physicians. Each of these disciplines bring a unique perspective and scope of practice, and more often today, they are collaborating to provide patient-centered care in healthcare settings. The authors of this article include an arts-in-health program director/artist, a creative arts therapist, a nurse/nursing scholar, and a physician. This array of disciplinary perspectives serves to provide clarity in regard to the scope of practice of artists working in clinical environments and to interpret the study data from a broader range of perspectives.

### 1.5. Medical Perspective

The traditional healing practices of Indigenous Western, Eastern, and other world cultures embraced the arts as a potent healing force millennia before modern medicine emerged 400 years ago. The Cartesian split between mind-spirit and body, and Newton’s “virile son”—science—reduced human beings to easily comprehensible machines [53]. Since some of these traditional practices (often called complimentary, alternative, or integrative medicine) have regained their firm foothold in biomedicine, their multidimensional concept of physical, psychosocial and socioeconomic wellbeing has become an important research endpoint for new medical treatments.

The medical profession cannot integrate the breadth of knowledge and skills needed to offer comprehensive healthcare when working in isolation. Yet physicians are not trained specifically to be part of interdisciplinary teams, to solve problems collectively, or to research the use of interprofessional medicine to advance human health [54]. A hundred years ago, the Flexner Report [55] dictated that medical education and research must adhere strictly to mainstream science. The phrase “interprofessional care team” would have baffled a general medicine physician in the 1930’s, and even in the 1950’s. However, a tipping point came when scientist-novelist C.P. Snow [56] condemned the schism between art and science, with its damning effects on our whole civilization. “Not to have read ‘War and Peace’…is not to be educated, but so is not to have a glimmer of the Second Law of Thermodynamics” [56] (p. 3).

The pendulum shift in healthcare since then is a response to global recognition of social determinants of health [57] and patient-centered care, led not by doctors but by other health professionals allying to create today’s interprofessional teams. Arts in health is an exemplar of integrating many aspects of health into care, defining health as “a positive concept emphasizing social and personal resources, physical capacity, healthy life-styles, and wellbeing” [58]. The UK government’s comprehensive report, Creative Health [22], is the outcome of a two-year inquiry into the place of art in interprofessional healthcare. Among their recommendations are that general practice physicians use “arts-on-prescriptions” for art for their patients. Artlift, a UK charity, showed in cost-benefit analysis that patients working with artists for six months had a 37% lower demand for general practice physician appointments and 27% reduction in hospital admissions—a net saving to the NHS of £216 per patient [22]. Today, these powerful trends are impelling doctors to integrate interprofessional health teams, including artists, into their teaching and practice.

### 1.6. Nursing Perspective

From the profession’s first beginnings, Florence Nightingale contended that nursing is an art. In keeping with this view, the contemporary nursing theorist, Jean Watson, describes nursing as an art and a science that expresses caring from the holistic view of the mind, body and soul or spirit [59]. In nursing care, the arts are deliberately utilized as a way to attend to the emotional and spiritual needs of patients [59,60]. As Watson explains, “The artistic expression of nursing includes attentional auditory, visual, sensory, olfactory, and tactile art or acts” [61] (p. 16). Traditionally, nurses spend the most time interacting and caring for patients and often develop a deep understanding of patient and family needs and desires [62]. The challenge for nursing has always been to attend to mind, body and spirit while providing care in an institutional setting.

Historically, nurses have worked together with artists to create programs that provide holistic attention and enhance patient outcomes. As the coordinators of care and patient advocates, nurses relate the need for humanistic care that can be delivered by other professionals. Nurses have been essential to the success of early art in health programs and served as researchers in studies related to the arts in health [44,63].

Many nurses advocate for inclusion of artists in the interprofessional team and communicate the role of artists to other professionals, patients and families, and healthcare administrators [44,63]. Nurses have reported that the arts are a highly-valued support for nurses [44]. With a shortage of nurses globally, nurses have embraced artists to provide cultural care and messaging to assist with health literacy activities; and to assist in care they are unable to give [23,44].

However, there is a need to educate nurses about the diverse roles that artists have within the health care arena and the educational background for each of these roles. Given that the discipline of arts in health is relatively new, nurses may not understand the education and training standards for professional artists in healthcare. As a result, the professionalism of artists may not be taken as seriously by other members of the interprofessional team. This decreases the perceived value of professional artist roles, particularly in light of the high level of education required by other health professions. Therefore, it is imperative to communicate the unique role of artists and their effect on patient outcomes and quality.

### 1.7. Creative Arts Therapies Perspective

Historically and presently, interprofessional collaboration is at the core of practice in the creative arts therapies which, according to the National Coalition for Creative Arts Therapies (NCATA) include music therapy, art therapy, dance/movement therapy, drama therapy and poetry therapy. Kaye and Hall [64] discuss the impact and value of the arts therapies on multidisciplinary teams, such as a palliative care team. Creative arts therapists, as clinicians, work closely with the healthcare team to deliver patient-centered care in both clinical and community health settings. Creative arts therapists actively participate in treatment planning including goal setting, assessment, intervention and service delivery, and re-evaluation of progress toward treatment goals. In addition, healthcare providers such as physicians, nurse practitioners, and nurses as well as physical and occupational therapists and child life specialists regularly make referrals to creative arts therapists to complement and enhance treatment goals and to address the physical, cognitive, emotional and/or psychosocial needs of the patient and their family members. Creative arts therapists are frequently called upon by the interprofessional team to address concerns such as psychological well-being, behavioral strategies, or cognitive functioning.

**Figure 1:**
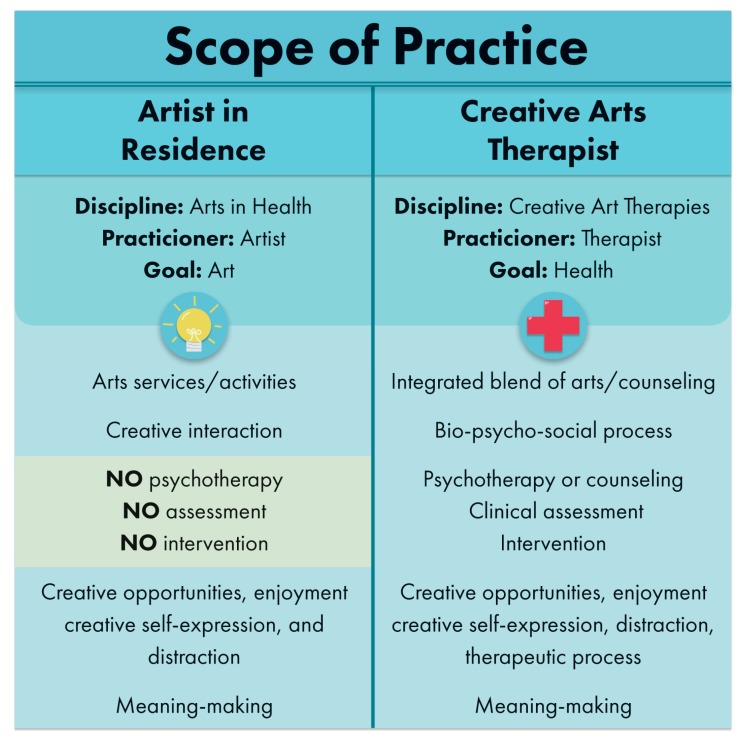
Scope of Practice.

Collaboration among creative arts therapists and artists in residence working in healthcare can enhance the interprofessional team by providing a spectrum across which the arts may be utilized for patient care and comfort. When both creative arts therapists and artists are available on the clinical team, a wider and more comprehensive range of creative tools, approaches and services are available to patients, family members, and the interprofessional team. To optimize engagement of these offerings, education may be provided to the interprofessional team as to what specific modalities are available to patients and in what circumstances each modality best fits. Figure 1 below, adapted from Imus [65], highlights distinctions in scope of practice between artists and creative arts therapists in healthcare.

## 2. Materials and Methods

A secondary analysis of a data set collected in 2014 was conducted to look for evidence of interprofessionalism among nursing and arts staff on an in-patient medical surgical unit. Arts staff included a unit-based artist in residence, artists in residence who served the unit by referral, and volunteer artists supervised by the artist in residence. No creative arts therapists worked on the unit prior to or during the study period. The qualitative study used a deductive approach to the analysis of individual structured interviews. The 2014 interviews were designed to assess the impacts of arts programming on job satisfaction, stress, unit culture, support, quality of care, and patient outcomes on a short-term medical-surgical unit. The original study used a qualitative cross comparison grounded theory methodology to analyze narrative inquiry data. The results of the study were subsequently published [44]. All study participants gave their informed consent for inclusion before they participated in the study. The study was conducted in accordance with the Declaration of Helsinki, and the protocol was approved by the University of Florida Institutional Review Board (project number 350-2010).

The secondary analysis, conducted in 2017, included a review of the literature on interprofessionalism and a directed content analysis [66] of four of nine questions included in the original interview. All 31 members of the unit's nursing staff participated in the interviews. The review of the literature identified 11 themes that were derived from current and seminal articles on interprofessionalism in healthcare and deemed to be potentially relevant to the analysis. These themes fell under four domains: values and ethics, roles and responsibilities, interprofessional communication, and teams and teamwork. These domains were identified in the panel report, Core Competencies for Interprofessional Collaborative Practice, published by the Interprofessional Education Collaborative [5]. Within these domains several competencies are identified. Specific competencies were selected as themes for the data analysis (see Domains 1–4 in Table 1, below).

In keeping with best practice recommendations for content analysis, a team of six coders (one principle investigator and five others) coded the data [67]. A codebook that listed and defined the eleven themes was developed for the directed content analysis. Research team members read the code book and discussed each theme to establish a mutual understanding of the identified domains and themes. In order to establish intercoder reliability, members of the team initially completed individual directed coding of the first 20 narrative responses to the first interview question. The team coded for the identified themes, and also identified new themes using a descriptive coding approach [68]. After independently coding, the group convened for discussion and consensus building. This cycle was repeated until intercoder reliability and interrater reliability of >80% were established [69]. The codebook was then finalized and applied to the directed content analysis of the full data set. The team undertook individual coding of 30 responses to each of four open-ended interview questions, and engaged in consensus building for each response in order to establish the master code list. This process included memo taking to track developing interpretations of the data. The master code list was then analyzed both quantitatively and qualitatively.

## 3. Results

The directed content analysis identified the presence of ten of 11 identified themes in the data, representing each of four domains identified in the literature, and their themes. Coding of the raw data resulted in the emergence of a fifth domain, quality of care, and two additional themes. In total, 13 themes were identified. Codes were applied to 58 of 120 responses (48%). No relevant codes were identified in the other 62 responses. Table 1 presents the identified domains and themes, along with their occurrences in the data set.

### 3.1. Domain 1: Values and Ethics

Themes from the values and ethics domain were quite prevalent in the data, with the relationship theme being the most commonly coded theme (*n* = 41). In most cases, the mutual respect component of this theme triggered the coding. Nursing staff members’ comments consistently reflected their respect for the arts as a complementary and helpful discipline and of artists as members of the care team.

“I find it helpful and I don't think it should ever stop!”

“I just appreciate what they do, and… this is a tough floor to work on because there’s a lot of total care patients.”

“If a patient is happy, they're going to heal faster. I feel like it works”.

Cooperation was nearly as prevalent with 31 comments indicating interdisciplinary collaboration between nurses and artists, and recognition on the part of nurses that there were resources beyond medicine that could aid their patients.

“…you have a resource here when you feel like the patient needs a different therapeutic outlet, that you have something that you can turn to.”

“I feel that I have other resources I can call on, when I have patients that need distraction, need something other than just nursing care.”

### 3.2. Domain 2: Roles/Responsibilities

The roles/responsibilities domain was highly represented in the data, including “competencies” (*n* = 31) and “expertise” (*n* = 21). Staff highlighted how artists enhance patient care and how their expertise complements the nursing staff’s own clinical and professional expertise. The role of artists was identified as unique and complementary to that of clinical staff.

“They do what we can’t.”

“It makes you more aware of other ways that you can be more creative in how you're impacting the care that you're giving the patient.”

“I think it gives me an alternative where if I know someone's bored, and they do want to do some drawing or painting, that I have an option.”

Respondents noted the positive distraction that art provides for patients as significant, especially in regard to pain management, enhancement of the overall atmosphere, lifting energy and providing an emotional outlet. Comments frequently highlighted the importance of the arts and artists as resources that extend the care that nursing staff members provide directly.

“I think it’s a help to the patient, especially with pain. It takes the patients’ minds off the pain and focuses it on other things”.

“If they’re busy doing something, it’s better than giving them medication. A lot of [patients] just need some diversion.”

Respondents also recognized that the art program and artists help them to address the emotional needs of patients, and to “extend” the care they physically provide by referring artists to work with patients. They also described a sense of accomplishment and satisfaction in the care they provide as a result.

“If you see your patient happy and they’re satisfied with what you’re giving them plus the arts in medicine helping us, it's a great feeling”.

Description of the art program and artists as “resources” for extending care was a significant finding, and clearly represents interprofessional teamwork across the two disciplines.

“I feel that I have other resources I can call on, when I have patients that need distraction, need something other than just nursing care.”

“We don't have the resource, but Arts in Medicine has the resource and they provide it to the patient. I think it’s a help to the patient, especially with pain”.

“Having that additional resource here, it's always helpful to the nurses.”

Additionally, nursing staff members described feeling that the work of artists provided them with a direct means for relaxation and enjoyment (i.e., when they hear music or see a patient making art). The relaxation and enjoyment were derived both from direct exposure to the arts as well as from the effects of the arts on their patients (the “happy patient/happy staff effect” is discussed below).

“It makes me more calm and I don’t get as worked up.”

“I know that [the nurses] like to hear [the music] because there's a lot of stress on the floor and it's nice to listen to [it]. I love that personally. It helps relax [us].”

“I love the artists. It helps me have a better day—especially when they come around with the flute and I mean it's therapy for me, I feel like, just as much as the patient.”

Respondents commonly articulated the value of the arts and artists in providing emotional support, advocacy, and attention for patients who have particularly high needs in medical-surgical care areas. While nursing staff often feel unable to provide this level of attention, they are grateful that an artist can provide creative engagement that can address a patient’s broader needs.

“It’s someone else that is an advocate for [the patient].”

“It reminds me that we are not just here to take care of the patients physical needs. They also have emotional needs. It reminds me that these patients need a little bit of that too.”

“I think it's just a very positive thing for the patients. [The presence of the artist] lets the patients know that we care for them and that she’s got something for them to do.”

The presence of artists on the interprofessional care team was noted consistently as an enhancement to patient care. The skills and abilities of artists and care providers were noted as complimentary.

“I think it's a better quality of care because, like I said, the patients are more relaxed”.

“… it helps alleviate some of the worry, you know, ‘well what can I do to entertain this patient’? Because I can't always entertain them all the time, so it helps to have them distracted by something else.”

“It's something to do to divert their pain. It helps me. And you know, you don’t have to give them pain medication”.

### 3.3. Domain 4: Teams and Teamwork

The domain of “teams and teamwork”, touched upon the impact of the arts on informing care decisions and patient-centered problem solving. Nurses described how they integrated the program into their clinical practices by engaging artists and the arts with clinical outcomes in mind.

“It can help entertain them, distract them, and re-direct them. So it assists me that way because someone else is spending that one on one time with them.”

“It takes the patients’ minds off of the pain and focuses it on other things. They’re able to relax which allows us to be able to relax because they're focused on other things. So it helps us.”

The impact of the distraction provided by arts engagement was a common theme in the data.

“It helps me because if they come in there, especially if my patients are in pain, and they come in, and they do an activity… We don't get to do that, like personal [attention] as a nurse, we don't get to do that much with the patients.”

“It is an excellent help. In pain, of course you don't want to be like… you can always bring them pain medication. But some of them just need some diversion. That’s the thing, they keep on watching the clock, but if they’re busy doing something, it’s better than giving them medication. A lot of them just need some diversion.”

Nurses also articulated integration of the arts and artists into their clinical practice, in general, and how the presence of the arts impacts their thinking in regard to clinical care.

“It's integrated into how I work”.

“It helps me to be creative and resourceful.”

“It makes you more aware of other ways how you can be more creative in how you're impacting the care that you're giving the patient.

### 3.4. Domain 5: Quality of Care

The theme of quality of care improvement was found 26 times in the nurse’s responses. The nurses often commented on how helpful it is to have the arts as an option, particularly for keeping patients occupied, which they view as an enhancement in regard to relaxation for both patients and staff, enhancement of the environment of care, and a more holistic approach to care that attends to a patient’s social and emotional needs as well as their physical needs.

“I think it's a better quality of care because, like I said, the patients are more relaxed.”

“[The arts] create a more joyful, positive, homey feel for [patients]. They are happier to be here than when there weren't extra activities around for them.”

“I think it's improved [the quality of care]. We know [the art program] is there, we're more likely to recommend or try to seek it out, knowing that it's available.”

“I think with [the art program], [the unit] gives excellent care for the patient”.

“I think [with arts in medicine] it's a better quality of care.”

The nurses also noted how the art program enhanced their own work lives, which has a direct impact on the quality of the care they can provide to patients.

“Oh my God, it really has relieved my stress. It has. It’s great.”

“It makes me love being here and working with my patients. It really does. It has a big impact.”

### 3.5. Happy Patient/Happy Staff Effect

The theme of “happy patient, happy staff” was identified eight times in the survey responses. Members of the nursing staff indicated that the arts and artists help to create a more positive hospital experience, as well as positive distraction and enjoyment for patients. Many of the nursing staff members also indicated that when patients are happy, it makes the nurse’s jobs easier, more enjoyable and more meaningful.

“I think it raises the morale of the patients. As long as we have happy patients, the staff will be happy.”

“It makes me relax as well. If I see my patient relaxed and not stressed, it makes me happy and relaxed in my 12-h shift. Indirect effect. If I have happy patients and they’re not stressed out for the whole day, then it makes me happy. Kind of fulfillment for the whole 12-h shift, because you know that's a long shift so if you see your patient happy and they’re satisfied with what you’re giving them, plus arts in medicine helping us, it's a great feeling.”

“It does make my job easier if they're less cranky.”

“It takes the patients’ minds off of the pain and focuses it on other things. They’re able to relax, which allows us to be able to relax because they're focused on other things.”

“When you support a patient, you’re supporting the staff.”

## 4. Discussion

Our findings suggest that the presence of artists on interprofessional teams may enrich the quality of healthcare by establishing a culture in which creativity and interdisciplinary collaboration are more valued and exercised. In keeping with recommendations offered by Pullon [11], findings of this study suggest that opportunities for regular communication and collaboration across care disciplines should be facilitated in as many routine ways as possible. The study suggests that collaboration with artists provides regular opportunities for such communication and also positively expands the care provided by the interprofessional care team. The findings also suggest that developing an interprofessional culture, including cross-professional respect and communication, can support the development of routine and sustainable interprofessional collaborative patient-centered care.

Nurses who participated in this study described how the arts contribute to a more creative environment in which they found themselves recognizing that they could look for solutions to challenges outside of their own discipline. The presence of the arts and artists on the unit also reminded nurses that creativity is in itself an asset in problem-solving, and stimulated more creative and interdisciplinary approaches to problem-solving.

Some areas of patient need, such as emotional need, identified by nurses in this study suggest that both artists and creative arts therapists are important members of interprofessional care teams. While the interaction and activities provided by artists may address some emotional needs very generally (i.e., a need for social interaction), they cannot address specific emotional needs on a clinical level.

From the perspective of clinical care providers, artists provide a form of patient advocacy and surveillance that improves overall care. While artists are not clinical care providers, they do attend to patients and they often participate in dialogue with clinicians that gives deeper insight into the strengths and needs of patients as whole individuals. Artworks created by patients also provide clinicians with a deeper understanding and appreciation of their patients as whole individuals, and can sometimes provide information about a patient’s values and viewpoints, personal experiences, and interests that can guide more patient-centered care.

While this study highlighted appreciation among nurses for artists in interprofessional care teams, it also brought forward the perspective—from nurses—that physicians may not share the same level of appreciation for this complement to care. The authors have considered gender as a variable in this finding. Globally, approximately 90% of nurses are women, and women compose greater than 90% of trainee nurses and approximately 80% of artists in healthcare as well [70,71]. Indeed, 100% of the nurses interviewed in this study were women. While more physicians have historically been men, the women’s movement has compelled an about-face in the gender of physicians over the past two decades. Today, 50% of US medical school enrollees are female. As such, women may be leading the interprofessionalism movement and the integration of artists into interprofessional teams, as this integration is largely due to nurse-artist alliances. Further research would be warranted on this topic. Additionally, further research that explores the perspectives of artists, physicians, creative arts therapists, and other interprofessional team members would be of value.

The arts are a highly cost-effective and low-risk component of healthcare today. Healthcare institutions are increasingly employing artists to work with patients and staff to impact the physical environment and culture of care. As a discipline, the arts are highly cost-effective and safe compared to other healthcare disciplines. Well-trained artists are adept in the areas of human engagement, communication, and expression. With professional artists as facilitators, the arts are providing healthcare with new avenues for enhancing patient-centered care, patient and staff satisfaction, and interprofessional collaboration.

However, healthcare is indeed a complex adaptive system that is perpetually on the edge of chaos [72]. Chaos and complexity science theories are increasingly being applied to interprofessional teams in healthcare. The recognition that creativity thrives in the space between stability and chaos is driving interprofessionalism and making space for creativity. Healthcare systems and the interprofessional teams are more acculturated today to disruption and the order that emerges from change; and inherent in the challenges of complexity are opportunities for creativity [73]. Inclusion of artists in the interprofessional team expands the concept of the team to include other disciplines, expertise and views, enhancing opportunities for creative problem-solving. Artists can also serve to dissolve the divisions in healthcare teams that stem from the heuristics of the medical notions of curing and nursing notions of caring [74].

The 5th-century Hippocratic Oath, still affirmed by new American physicians, states, in part: “I will preserve the purity of my life and my arts… If I keep this oath faithfully, may I enjoy my life and practice my art, respected by all humanity…” [73]. Similarly, Watson [75] asserts that nursing is the art and a science of caring. Today, the presence of artists and the arts in healthcare environments is reminding healthcare professionals in all disciplines that caring is an art. Their presence is also contributing to greater levels of interprofessional caring and a more holistic provision of care. Despite the still pervasive influence of Cartesian and Newtonian thinking on modern healthcare, the arts are helping to shape a new healthcare culture in which interprofessional care is offered as no less an art than a science.

## 5. Conclusions

Our findings suggest that artists offer a valuable component to interprofessional healthcare teams, with several dimensions of interprofessionalism being strengthened by artists, including cooperation, teamwork, and the breadth of team competencies and expertise. The environment and quality of care provided by primary clinical staff members is enhanced by artists, who offer holistic attention, distraction, and enjoyment, which creates a positive and enjoyable experience for patients and staff alike. Inclusion of professional artists in interprofessional healthcare teams may lead to higher levels of support for patients as well as for staff, and assist care teams in achieving patient-centered care goals.

## Figures and Tables

**Table 1:** Study Domains and Themes and Their Occurrence in the Data.

**Domain 1: Values and Ethics**	**In Data**
Cooperation: Cooperation among those who provide care recognizing the multidisciplinary nature of health delivery systems	31
Relationships: the quality of cross-professional exchanges, and interprofessional ethical considerations in delivering health care, including mutual respect	41
Values: Consistent demonstration of core values evidenced by professionals working together, aspiring to and wisely applying principles of altruism, excellence, caring, ethics, respect, communication and accountability to achieve optimal health and wellness in individuals and communities	10
**Domain 2: Roles/Responsibilities**	
Competencies: Use unique and complementary abilities of all members of the team to optimize patient care	31
Roles: Communicate one’s roles and responsibilities clearly to patients, families, and other professionals	0
Expertise: Engage diverse healthcare professionals who complement one’s own professional expertise, as well as associated resources, to develop strategies to meet specific patient care needs	21
Roles: Explain the roles and responsibilities of other care providers and how the team works together to provide care	7
Expertise: Use the full scope of knowledge, skills, and abilities of available health professionals and healthcare workers to provide care that is safe, timely, efficient, effective, and equitable	6
**Domain 3: Interprofessional communication**	
Effective communication: Communicate consistently the importance of teamwork in patient- centered and community-focused care	12
**Domain 4: Teams and Teamwork**	
Role and competencies of the team: Integrate the knowledge and experience of other professions— appropriate to the specific care situation—to inform care decisions, while respecting patient and community values and priorities/preferences for care	17
Role and competencies of the team: Engage other health professionals—appropriate to the specific care situation—in shared patient-centered problem-solving	14
**Domain 5: Quality of Care**	
Quality enhancement: The process of taking deliberate steps at the institutional level to improve quality	26
Happy patient happy staff effect: Increasing nurse happiness by making work easier, enhancing nurse/patient communication, and increasing fulfillment with caring as a result of patients having a more positive overall experience	8
